# Precision medicine in cats—The right biomedical model may not be the mouse!

**DOI:** 10.1371/journal.pgen.1009177

**Published:** 2020-12-08

**Authors:** Leslie A. Lyons

**Affiliations:** Department of Veterinary Medicine and Surgery, College of Veterinary Medicine, University of Missouri, Columbia, Missouri, United States of America; University of Helsinki, FINLAND

On the best of days, using state-of-the-art genetic approaches involving whole genome and whole exome sequencing (WGS/WES), geneticist have only approximately a 50:50 chance of rapidly identifying variants causal for health and developmental abnormalities in humans [1]. Variants of unknown significance (VUS) now plaque WGS/WES studies, and a plethora of bioinformatic approaches have been developed to predict VUS pathogenicity [2]. One common approach to define the function of a VUS is to create the animal model, hence produce a genetically modified organism focused on the VUS of interest. For mammalian biology, rodents are the most easily genetically modified species, with porcine models developing quickly [3,4]. Genome editing of induced pluripotent stems cells supports VUS studies by creating the “disease in a dish” [5,6]; however, information from other species, comparative genetics, remains an invaluable tool to decipher VUS physiological effects, thereby influencing their priority for investigation. The research by Graff and colleagues, “***PEA15* loss of function and defective cerebral development in the domestic cat**,**”** is a strong example of when the murine model just does not rise to the challenge [7], and the value of other species models is recognized.

Based on the analysis of primary astrocyte cultures from knockout mice, *phosphoprotein expressed in astrocytes-15* (*Pea15)* has been known for decades to be expressed in astrocytes and normally functions to suppress tumor necrosis factor alpha (Tnfα)-induced apoptosis in these cells [8]. However, mice with *Pea15-*targeted mutations have normal brain size and morphology, contrary to a newly defined neurological model in domestic cats [7,9]. Thus, *PEA15* was not strongly implicated in brain development. The Graff and colleagues study is an outstanding example of the continued importance of spontaneous conditions in large animal models, specifically the domestic cat. Hundreds of companion animals have been identified with DNA variants in genes that also cause similar human diseases (Table 1) [10]. Recent WGS studies in domestic cats have implicated causal variants in novel genes, including *KIF3B* variants causing retinal degeneration (OMIA 002267-9685), *UGDH* causing disproportionate dwarfism (OMIA 000187-9685), and *GDF7* associated with another brain dysmorphology (OMIA 000478-9685), all diseases with undiagnosed human patients [11–13]. New models for neuronal ceroid lipofuscinosis (OMIA 001962-9685; OMIA 001443-9685) have further utilized WGS and now WES in domestic cats [14,15]. Intergenic structural variation (SV) and genome organization variation are becoming more recognized as keys to gene function. The importance of SVs in the cat is demonstrated by common hypomelanistic and amelaninistic phenotypes. White cats are one of the historical models for neurological studies, as a high percentage of all white cats have congenital deafness. *White* is a dominant trait in domestic cats caused by an approximately 700-bp insertion in intron 1 of *KIT*, which is a gene known to cause various white spotting phenotypes in different species and known in the development and migration of melanocytes from the neural crest [16,17]. Interestingly, a larger insertion of approximately 7 kb at the same intronic position causes a phenotype with a lesser degree of white, the phenotype known as *Spotting*. A large 20-kb duplication of the lysosomal traffic enzyme (*LYST*) is another SV in cats, causing yet another lysosomal storage disease associated with neurological deficits [18]. The increased vigilance of feline health by cat owners and the maturation of genetic resources are supporting the reemergence of cats as biomedical models, particularly for neurological studies.

**Table 1:** Online Mendelian inheritance in animals: Biomedical models for human disease in non-rodent mammals.

	Dog	Cattle	Cat	Pig	Sheep	Horse	Chicken	Rabbit	Goat	Other	TOTAL
Total traits/disorders	776	545	357	280	256	240	222	98	88	674	3,626
Mendelian trait/disorder	358	254	113	86	110	59	131	58	18	266	1,518
Mendelian trait/disorder; likely causal variant(s) known	286	161	80	39	55	46	51	11	14	139	898
Likely causal variants	417	217	128	47	70	97	66	14	25	120	1,219
Potential models for human disease	462	220	222	128	115	132	51	54	40	350	1,807

Accessed 2020 Sep 4 [10].

Historically, the cat has been a favored model for neurological studies. Since rodents lack gyri and sulci, abnormalities in cerebral cortical proliferation and folding are challenging to study in laboratory mice. Lysosomal storages diseases often lead to neurological deficits, in which cats are highly valued models and are used in therapeutic trials [19]. In Graff and colleagues, ironically, cats with an autosomal recessive cerebral dysgenesis phenotype were identified within an Auburn cat colony established for lysosomal disease investigations. The affected cats had a 45% decrease in overall brain weight, defective gyrification, expansion of astrocytes, and a loss of mature oligodendrocytes and white matter; however, their gross appearance and behavior are not significantly abnormal. Thus, the cat is the right biomedical model for the right disease, suggesting an underlying pathophysiology and a developmental process that is unique to animals with gyrencephalic brains.

The Graff and colleagues research also demonstrates the maturity of WGS in domestic cats and the utility of the cat variant database [20]. The 99 Lives Cat Genome Sequencing Consortium now encompasses the genetic variation from over 300 domestic cats, and its use for precision medicine is maturing [20]. In the cerebral dysgenesis study, WGS of eight affected and six obligate carrier cats identified an area enriched for candidate variants in a distal 5-Mb region on cat chromosome F1q. Genotype by sequencing reduced the region to a 1.3-Mb haplotype with 337 variants that were private and not present in the 99 Lives dataset. The *PEA15* coding sequence variant (XM_023247767.1:c.176delA, XP_023103535.1:p.(Asn59fs)) had the highest Combined Annotation Dependent Depletion (CADD) score, predicted a frameshift and early truncation likely leading to nonsense-mediated decay, and the gene is highly expressed in the brain. RNA sequencing (RNA-seq) and immunohistochemical analysis revealed astrocytosis. Expression levels of *PEA15* as assessed by RNA-seq from the cerebral cortex of adult cats revealed a 59% reduction in homozygous affected animals, consistent with nonsense-mediated decay. Western blot analysis with a polyclonal antibody against the carboxyl-terminal amino acids of human *PEA15* indicated that a 15-kDa band present in normal brain extracts was absent in brains from affected cats. Together, these studies strongly implicate the *PEA15* variant as causal for the autosomal recessive cerebral dysgenesis, causing the death of neurons accompanied by increased proliferation of astrocytes, leading to abnormal organization of neuronal layers and loss of white matter.

A vast majority of the genes in the human body have direct homologs in all mammals, including domestic cats [21]. *PEA15* is highly conserved across mammalian species; however, no gene-specific pathogenic variants are defined that are associated with brain malformations. Compared to humans, cats have higher sequence identity across their exome and higher protein homology than mice, suggesting many antibodies developed for human protein studies may be suitable for use in the cat, as demonstrated by the *PEA15* Western blot analyses in the cerebral dysgenesis study. Since cats have high genetic similarity and conserved genomic organization with humans, when physiological and developmental biology are also conserved, perhaps they can better decipher VUS for human studies than more traditional mammalian disease models.

How can researchers be more efficient in developing the right biomedical model for the right disease? Similarities and differences across species will define the biological effects due to perturbations in gene order, distance between genes, and the role of distant regulatory elements. Overall, genomic organization similarities and differences have not been strongly considered as important factors for biomedical models, perhaps it’s time to reconsider gene synteny across species when exploring SV and gene regulation? Assisted reproduction is well developed in the domestic cat and has been used to resurrect neurological disease models [18]. More focus and support for genome editing in cats will help produce cat models when rodents and porcine are not appropriate. Different animal models have various costs and benefits; the advances in WGS/WES in companion animals will lead to additional novel discoveries useful as biomedical models for human diseases. Additional support for cat genomics and genome editing could lead to effective, feline-based biomedical models that fill an important void for VUS interpretation, targeted therapeutics, and translation medicine.
